# Oxidant–antioxidant status and assessment of cardiovascular morbidity in Pan Masala containing Tobacco users: a cross-sectional study

**DOI:** 10.1186/s13104-018-3832-5

**Published:** 2018-10-12

**Authors:** Sagar Khanal, Seraj Ahmed Khan, Dharnidhar Baral, Sanjeeb Shrestha, Nirmal Baral, Madhab Lamsal

**Affiliations:** 1 Department of Biochemistry, Lumbini Medical College, Palpa, Nepal; 2 SPH and Community Medicine, BPKIHS, Dharan, Nepal; 3 Department of Biochemistry, Gandaki Medical College, Pokhara, Nepal; 4 Department of Biochemistry, BPKIHS, Dharan, Nepal

**Keywords:** Pan Masala containing Tobacco, Malondialdehyde, Vitamin C, Vitamin E

## Abstract

**Objective:**

Pan Masala containing Tobacco (PMT) use contributes significantly to the overall world tobacco burden especially in south Asian country like Nepal. Oxidative stress caused by it may leads to cardiovascular disease, peripheral vascular disease, hypertension, etc. Therefore, this work proposes to study the antioxidant and oxidative stress along with cardiovascular morbidity in PMT users.

**Results:**

Hundred PMT users and 80 non-user controls with age and sex matched were enrolled. There was a significant difference in blood pressure, albumin, uric acid, vitamin C, vitamin E, malondialdehyde (MDA), total cholesterol, triglycerides, low density lipoprotein cholesterol between the two groups (p < 0.001). We observed statistically significant (p < 0.001) decrease in antioxidant and increase oxidative stress in PMT users. Duration and quantity of PMT user were significantly associated with the MDA level.

## Introduction

Most common form of smokeless tobacco (ST) use in south Asian country, is PMT, which is commonly known as “Gutkha”. It is a significant burden of the overall world tobacco problem in the region including Nepal. Besides PMT there is a great diversity of smokeless tobacco products and their use patterns across the globe [1]. Global Adult Tobacco Survey reports that 3 billion peoples across 16 countries use tobacco products in different forms [2]. In a low income country like Nepal, with low education rate, it is socially acceptable habit in nearly two third of the population [3–5]. In Nepal, an estimated 4.1 deaths per day are due to tobacco-related diseases, which accounts for 1500 deaths annually [4].

Consumption of PMT may play a contributory role in the development of cardiovascular disease, peripheral vascular disease, hypertension, peptic ulcer and fetal morbidity and mortality. Tobacco-specific alkaloids, including 4-(nitrosomethyl-amino)-1-(3-pyridyl)-butanon (NNK), *N*′nitrosonornicotine (NNK), *N*′nitrosoanabasine (NAB) and nitrosoanatabin (NAT) are noted carcinogens and also, it causes oxidative stress in the human body [6–8].

Recent studies on cell culture have demonstrated that long-term usage of PMT and tobacco related products are the major contributor of free radicals [9]. The body tightly regulates pro-oxidants and antioxidant level for maintaining vital cellular and biochemical functions. Any alteration in the balance could result in increase oxidant against the capacity of antioxidant leading to oxidative stress, which have deleterious effects on the body.

Our body have evolved complex mechanism of antioxidants like catalase, superoxide dismutase (SOD), reduced glutathione, vitamin C, vitamin E, bilirubin, uric acid, albumin etc., to scavenge reactive oxygen species (ROS). Studies have shown depleted antioxidant enzymes and vitamins causing oxidative stress in tobacco users and smokers [7–9]. Researchers uses malondialdehyde (MDA), an end product of lipid peroxidation by ROS, as a marker of oxidative stress [10].

Many evidences support the role of oxidative stress in the development and progression of cardiovascular disease, which is the major cause of mortality and morbidity worldwide [11–14]. Oxidative stress not only results in oxidation of low-density lipoprotein (LDL), which is a crucial step in the pathogenesis of atherosclerosis, but also causes vascular dysfunction by promoting pathological growth of vascular smooth muscle cells (VSMC) and fibroblasts, triggering DNA breaks, causing apoptosis of endothelial cells, and increasing platelet adhesion, all of which contribute to the development of atherosclerotic disease [15–17].

The prevalence of cardiovascular risk is different in different modes of tobacco use. In this study, we aim to examine different oxidant and anti-oxidant markers as well as lipid profile in subjects consuming PMT and evaluating the risk factor for cardiovascular disease.

## Main text

### Methods

This cross sectional study was conducted among the PMT users and non-users control from Dharan municipality in the Department of Biochemistry at B.P. Koirala Institute of Health Sciences, Dharan, Nepal.

A total of 180 subjects were recruited in the study, out of which 100 were PMT users and 80 were age and sex matched non-user control subjects. The snowball sampling technique was used. Presence of any self-reported diagnosed case of cardiac, renal or hepatic disease, any current treatment for cardiac or blood pressure related morbidities and history of heavy alcohol or recreational drug use and those not willing to take part in the study were excluded.

Following the standard aseptic technique 5 ml blood was collected in plain and Ethylenediaminetetraacetic acid (EDTA) vials, after taking the consent from the participants. Serum and plasma were separated by centrifugation at 3000 rpm for 10 min. Samples were stored at − 20 °C until the analysis.

A structured questionnaire including name, historic period, gender, stature, weight, SBP, DBP, tobacco, PMT chewing habits, duration of PMT use was filled up after getting the informed consent from the subjects.

MDA [18], vitamin C [19], vitamin E [20], albumin, uric acid, bilirubin and lipid profile (TC, TG, LDL-C and HDL-C) were estimated spectrophotometrically by measuring the optical density of standard and sample by Spectrophotometer 259 (Sherwood Scientific Ltd, UK).

Statistical analyses were performed using the statistical package for social sciences (SPSS-18). Normality of the data was tested by Kolmogorov–Smirnov test. Data were expressed as mean (SD), median, percentage and frequency. An independent samples t-test and Mann–Whitney U was used for comparing means of metric data. For correlation analysis Pearson’s correlation and Spearman’s rho was used. Multiple linear regression model was used to see the independent effect of variables on oxidative stress. Probability of significance was set at 5% level of significance.

### Results

The mean age of PMT user group was 37.94 ± 6.05 years whereas the mean age of non-user control group was 33.61 (7.57) years. The majority of the participants in both the groups were male. In PMT user the numbers of male and female was 87 and 13 respectively and 65 and 15 respectively in non-user control group. Majority of the participants in both the group were non-vegetarian and the difference in BMI was not significant between the groups (p = 0.078). The mean duration of the PMT use was 11.41 (7.0) and number of packets consumed per day was 6 (3, 13). PMT users and non-user control consumed less vegetables and fruits. In biochemical parameters except for bilirubin and HDL, all the others like albumin, uric acid, vitamin C, vitamin E, MDA, TC, TG and LDL-C showed a significant difference between the two groups (p < 0.001) (Table 1).

**Table 1:** General and biochemical parameters of the study participants

Variables	PMT users (n = 100)	Non users (n = 80)	p value
Age	37.94 (6.05)	33.61 (7.57)	< 0.001
Gender (n)			
Male	87	65	NS
Female	13	15	
Dietary habit (n)			
Veg	8	6	NS
Non-veg	92	74	
BMI (kg/m^2^)	24.91 (3.73)	24.0 (4.0)	0.078*
SBP (mmHg)	129.35 (12.67)	117.69 (8.41)	< 0.001*
DBP (mmHg)	90.35 (9.70)	79.50 (7.94)	< 0.001*
Vegetable (/week)			
< 7	85	69	0.813**
7	15	11	
Fruits (/week)			
< 7	87	68	0.700**
7	13	12	
Duration (years)	11.41 (7.0)	–	NA
Quantity (Pkt/day)	6 (3.13)	–	NA
Albumin (g/l)	41.90 (4.44)	45.09 (4.89)	< 0.001*
Uric acid (μmol/l)	333.98 (73.92)	405.30 (127.94)	< 0.001*
Bilirubin (μmol/l)	9.97 (6.40)	9.85 (6.07)	0.933*
Vitamin C (μmol/l)	58.18 (25.34)	90.05 (28.46)	< 0.001*
Vitamin E (μmol/l)	51.37 (16.59)	64.83 (22.58)	< 0.001*
MDA (nmol/ml)	8.26 (1.67)	4.40 (1.83)	< 0.001*
TC (mg/dl)	210.44 (44.7)	161.54 (31.78)	< 0.001*
TG (mg/dl)	157.18 (82.16)	122.14 (45.32)	< 0.001*
LDL-C (mg/dl)	132.71 (42.14)	92.49 (26.69)	< 0.001*
HDL-C (mg/dl)	45.50 (8.71)	47.33 (9.28)	0.177*

* Independent t test

** Chi square

p < 0.05 is considered statistically significant

In the correlation analysis, duration of PMT use, quantity of PMT, SBP, DBP, albumin, TC, TG (p = 0.008) and LDL-C were positively correlated with MDA level, whereas UA, vitamin C and vitamin E were negatively correlated with MDA and was statistically significant (p < 0.001) (Table 2).

**Table 2:** Correlation of the variables with MDA (n = 100)

Variable	Correlation coefficients (r)	p-value
Duration of PMT use in years	0.433	< 0.001*
Quantity of PMT use in pkt/day	0.414	< 0.001*
BMI (kg/m^2^)	0.029	0.694*
SBP (mmHg)	0.349	< 0.001**
DBP (mmHg)	0.309	< 0.001**
Albumin (g/l)	− 0.382	< 0.001**
Uric acid (μmol/l)	− 0.344	< 0.001**
Bilirubin (μmol/l)	0.078	0.301**
Vitamin C (μmol/l)	− 0.242	0.001**
Vitamin E (μmol/l)	− 0.380	< 0.001**
TC (mg/dl)	0.387	< 0.001**
TG (mg/dl)	0.196	0.008**
LDL-C (mg/dl)	0.358	< 0.001**
HDL-C (mg/dl)	− 0.073	0.331**

* Spearman’s Rho

** Pearson’s correlation

p < 0.05 is considered statistically significant

To investigate the independent effects of UA, bilirubin, vitamin C, vitamin E, age, duration of use and number of packets used on MDA level (oxidant marker) in PMT users, multiple linear regression analysis was performed. Model fit the data (p < 0.001) very well and explains 45.4% variance in the outcome variables. We observed that the duration of PMT use and quantity of PMT is independently associated with MDA level and is statistically significant (p < 0.001; p = 0.001) (Table 3).

**Table 3:** Multiple linear regression analysis for the effect of independent variables on MDA in PMT users

Variable	B	SE	t	p
Constant	7.183	2.175	3.303	0.001
Albumin	− 0.010	0.043	− 0.226	0.822
Uric acid	− 0.003	0.002	− 01.628	0.107
Bilirubin	0.013	0.020	0.662	0.510
Vitamin C	− 0.001	0.005	− 0.213	0.832
Vitamin E	0.002	0.008	0.238	0.812
BMI	− 0.032	0.035	− 0.916	0.362
Age	0.023	0.023	1.014	0.313
Duration	0.109	0.029	3.697	0.000*
Quantity	0.085	0.027	3.099	0.001*

*B* coefficient, *SE* standard error, *MDA* malondialdehyde, *PMT* Pan Masala containing tobacco, *BMI* body mass index, *SBP* systolic blood pressure, *DBP* diastolic blood pressure, *TC* total cholesterol, *TG* triglyceride, *LDL-C* low density lipoprotein cholesterol, *HDL-C* high density lipoprotein cholesterol

p < 0.05 is set as statistically significant

### Discussion

In the present study, we have evaluated the different oxidant and antioxidant markers and also the different cardiovascular risk factors like BMI, systolic, and diastolic blood pressure, lipid profile like TC, TG, HDL-C, LDL-C to see whether PMT users are more prone to CV risk factors. We found systolic and diastolic BP, TC, LDL-C and lipid peroxidation product MDA to be significantly higher in PMT users than healthy controls suggesting that there is increased oxidative stress in PMT users.

As regards to dietary habits, most of the subjects were non-vegetarian and most consumed vegetables almost every day (> 7 times/week). Most of the cases were of medium socioeconomic status, which was considered according to their lifestyle, education, etc. There was no any significant difference in BMI between the case and control. The systolic and diastolic blood pressure was significantly higher in PMT users. Our finding was in accordance to the study done by Gupta et al. [21]. This could be due to a more prolonged absorption of tobacco when chewed accompanied with more prolonged vasoconstriction.

Several studies have reported an increase MDA level in tobacco chewers as compared to non-chewers [22–25]. In the present study we found higher MDA level in PMT users as compared to non-user control group and was statistically significant. In correlation analysis parameters like SBP, DBP, duration and quantity of PMT use were positively and significantly related to MDA, pointing towards the increase vulnerability of the users to CVD. Multiple linear regression analysis reveals that duration and quantity of PMT use were independently associated with MDA. Suggesting to the fact that the longer the exposure to PMT the more is their oxidative stress which makes them at high risk for CVD.

The body’s defense mechanism uses antioxidants to combat the damage caused by free radicals. When body has excess free radicals and not enough antioxidants in the body, a condition known as oxidative stress occurs. This could potentially result in diseases, including cancer and heart disease. Our body is equipped with enzymatic (first line) and non-enzymatic antioxidants (second line) [26]. In the present study we observed significant decrease in antioxidants like vitamin C and E in PMT users. Similar findings were reported by Bhimwal et al. and Giraud et al. [27, 28]. Depletion of antioxidants is a risk factor for chronic diseases like cancer and coronary heart disease. Our result reported a negative correlation of MDA with various antioxidants in the body. This findings further enhance the load of oxidative stress on PMT users which eventually could result in CVD. Hence the risk factors for CVD in PMT users should be monitored on a regular basis to prevent and control the disease.

In the present study the TC, LDL-C, and TG were higher in PMT users when compared to non-user control group. Higher levels of cholesterol and TG in PMT users may be attributed to tobacco induced stimulation on the metabolism of free fatty acids in peripheral tissue. Similar findings have been reported earlier. A large population based study found that subjects who regularly used ST products had 2.5 times the prevalence of hypercholesterolemia compared with non-users of tobacco [29, 30]. Tobacco chewers showed significantly decreased levels of HDL cholesterol and significantly increased levels of LDL cholesterol and TG. However, in contrary, some study found no significant differences in total or HDL cholesterol between ST (snuff and chewing tobacco) product users and nonusers [31, 32], which is in accordance with our study. The altered lipid profile in tobacco chewers is frequent finding, and the reason to this pattern can be explained by the mechanism proposed by Brischetto et al. [33].

## Limitations

The cross-sectional design of the study fails to establish a temporality of association between PMT and CVD risk. Data can be only suggestive of causation. Therefore, a long term prospective study could be the suitable explanation to the shortcomings of our study.

## Abbreviations

PMT: Pan Masala containing Tobacco
MDA: malondialdehyde
TC: total cholesterol
TAG: triacylglycerol
LDL: low density lipoprotein
HDL: high density lipoprotein
CVD: cardiovascular disorder
SOD: superoxide dismutase
ROS: reactive oxygen species
VSMC: vascular smooth muscle cell
EDTA: ethylenediaminetetraacetic acid
SBP: systolic blood pressure
DBP: diastolic blood pressure
SD: standard deviation
BMI: basal metabolic index
ST: smokeless tobacco
TSNA: tobacco specific nitrosamines
